# Can surgical management of bone metastases improve quality of life among women with gynecologic cancer?

**DOI:** 10.1186/1477-7819-12-250

**Published:** 2014-08-05

**Authors:** Tao Ji, Ramez Eskander, Yifei Wang, Kunkun Sun, Bang H Hoang, Wei Guo

**Affiliations:** 1 Musculoskeletal Tumor Center, People’s Hospital, Peking University, Beijing 100044, China; 2 Department of Obsterics and Gynecology, Division of Gynecologic Oncology, University of California Irvine Medical Center, Orange, CA 92868, USA; 3 Department of Pathology, People’s Hospital, Peking University, Beijing 100044, China; 4 Department of Orthopaedic Surgery and Chao Family Comprehensive Cancer Center, University of California Irvine Medical Center, Orange, CA 92868, USA

**Keywords:** gynecological cancer, bone metastasis, surgery, quality of life, palliative treatment

## Abstract

**Background:**

The evaluation, counseling, and management of gynecologic patients with bone metastasis remain a challenge for clinicians. In order to critically evaluate the role of surgery, we retrospectively analyzed the records of 18 patients surgically treated for metastatic gynecologic tumors of bone, focusing on quality of life, local tumor control, and survival.

**Methods:**

Eighteen patients underwent surgical procedures for the treatment of bone metastases secondary to gynecologic cancer between September 2003 and April 2012. The primary cancer sites included the uterus (n = 10), the cervix (n = 5), and an ovary (n = 3). Patients were followed for an average period of 13.8 months (range, 2 to 34 months). A visual analog pain scale (VAS) and Eastern Cooperative Oncology Group (ECOG) performance status were evaluated both pre- and postoperatively.

**Results:**

The median survival time following diagnosis of bone metastasis was 10.0 months. The mean VAS score was 5.8 preoperatively compared with 2.1, 3 months after surgery. The mean pre and postoperative ECOG performance status grades were 3.1 and 2.3, respectively.

**Conclusions:**

The prognosis of gynecological cancer patients with bone metastasis is poor. Some patients had improvement in their quality of life after surgical intervention for bone metastases; however, novel integrated treatment modalities should be investigated.

## Background

Ovarian, uterine and cervical cancer will affect an estimated 80,000 women in the United States, with 27,000 deaths [1]. Improvements in surgery, radiation and the development of novel chemotherapeutic agents have led to prolonged survival and an increase in the prevalence of bone metastasis [2]. The incidence of bone metastasis secondary to endometrial cancer is reported to be 6 to 15% [3,4]. Bone metastases occur in approximately 1.2% of patients with ovarian cancer [5] and represent the third most common site of metastasis in cervical cancer, occurring in 1.1% of patients [6,7]. Traditionally, radiotherapy is considered the first line treatment in the management of metastatic bone lesions [8].

Traditionally, patients with bone metastasis have advanced stage disease, and the utility of surgical intervention is unclear. Some advocate minimal intervention and aggressive pain control, while others support more aggressive surgical intervention due to the unresponsiveness of these metastatic lesions to chemotherapy, radiotherapy and other noninvasive measures. The established indications for surgery include impending or existing pathological fractures, spinal cord compression, and pain, especially for patients who became resistant to radiotherapy [9,10].

The evaluation, counseling, and management of gynecologic cancer patients with bone metastasis remain a challenge for practicing gynecologic oncologists. Existing reports on surgical treatment and outcomes are of limited value since most are based on case reports with limited follow-up data [11-14]. In order to critically analyze the role of surgery in this patient population, we retrospectively analyzed the records of 18 patients surgically treated for metastatic gynecologic tumors of bone, focusing on quality of life, functional outcome, local tumor control, and survival.

## Methods

Following institutional review board approval, we conducted a retrospective review of patients undergoing surgical procedures for metastatic gynecologic tumors over a nine-year period (September 2003 to April 2012). The study is approved by the Ethical and Professional Committee of People’s Hospital. Informed consent was obtained from all the patients. Patients were identified from the institutional cancer registry and cross-referenced to the pathology database.

The median age at diagnosis was 55.0 years (range, 35 to 76 years). Primary tumor identification was based on pathologic diagnosis and correlated, when appropriate, to initial surgical specimens. Patients were followed for an average period of 13.8 months (range, 2 to 34 months).

Patient information is summarized in Table 1. The primary cancer sites were uterus (n = 10; two with leiomyosarcoma), cervix (n = 5), and ovary (n = 3). Twelve of the 18 patients who were referred initially received gynecological treatment somewhere else. Bone was the only site of identifiable metastasis in 15 patients. The mean interval from the primary diagnosis of gynecologic cancer to the detection of bone metastasis was 21.8 months (range, 0 to 48 months). Two patients were referred with acute partial paralysis before the primary tumor was identified. Patients included in this review were referred to our institution from various hospitals, and thus, up-front treatment for their primary gynecologic malignancy was not uniform.

**Table 1:** Clinical characteristics of 18 patients in current study

**Patient number**	**Age**	**Primary tumor**	**FIGO stage**	**Treatment for primary tumor**	**Interval to bone metastasis (month)**	**Site of bone metastasis**	**Other site of metastasis**	**Indication for orthopedic surgery**	**Therapy for bone metastasis after surgery**	**VAS (Pre/post-operative)**	**ECOG (Pre/post-operative)**	**Follow-up from orthopedic surgery (month)**	**Status**
1	59	EC	IVA	Chemo + RT	37	Sacrum		IP	RT	6/1	3/1	10	DOD
2	68	EC	IIIB	Surg + Chemo	9	Pubic bone		IP	CT	4/0	2/1	30	DOD
3	48	OC	IIIC	Chemo	22	Humerus		PF		5/1	2/1	8	DOD
4	75	OC	IVB	Chemo + RT	at diagnosis	Spine/T3		PF + SCC		4/1	4/2	6	DOD
5	51	EC	IVB	Surg + Chemo	19	Spine/T5	Lung	SCC		6/2	3/3	13	DOD
6	63	OC	IIIB	RT	8	Spine/L2,3		IP		8/4	4/3	11	DOD
7	55	CC	IIB	Surg + Chemo	16	Femur		PF	RT	4/3	4/2	15	DOD
8	55	EC	IIIC	Surg + Chemo + RT	26	Spine/L3		IP		4/1	3/2	28	DOD
9	45	EC	IVB	Chemo + RT	at diagnosis	Spine/T12	Liver	SCC		7/2	3/4	3	DOD
10	55	EC	IVA	Surg + Chemo	16	Spine/L3	Liver	IP		6/8	4/2	16	DOD
11	55	EC^❖^	IIA	Surg + Chemo	39	Femur		IP	CT	6/1	3/1	34	NED
12	58	EC	II	Surg + Chemo	48	Acetabulum*/L1		IP		8/3	4/4	4	DOD
13	46	CC	IIB	Surg + Chemo + RT	25	Pubic bone		IP		7/3	1/2	6	DOD
14	35	CC	IIIB	Chemo + RT	14	Sacrum*/Ilium		IP	RT	9/1	4/2	6	DOD
15	76	EC	IIIB	Surg + Chemo + RT	10	Spine/L3	Lung	IP	RT	6/4	3/4	4	DOD
16	56	EC^❖^	IB	Surg + Chemo	12	Femur		PF		4/1	4/2	24	AWD
17	46	CC	IIIB	Chemo + RT	20	Femur		PF	CT	5/0	3/1	20	NED
18	47	CC	IIB	Surg + Chemo + RT	27	Humerus*/Ilium		PF	RT	6/1	2/4	5	DOD

AWD, alive with disease; CC, cervical cancer; Chemo, chemotherapy; DOD, died of disease; EC, endometrial carcinoma; EC^❖^, uterine leiomyosarcoma; IP, intractable pain; NED, no evidence of disease; OC, ovarian carcinoma; PF, pathological fractures; RT, radiotherapy; SCC, spinal cord compression; Surg, surgery.

*Indicates the operation site.

**Figure 1:**
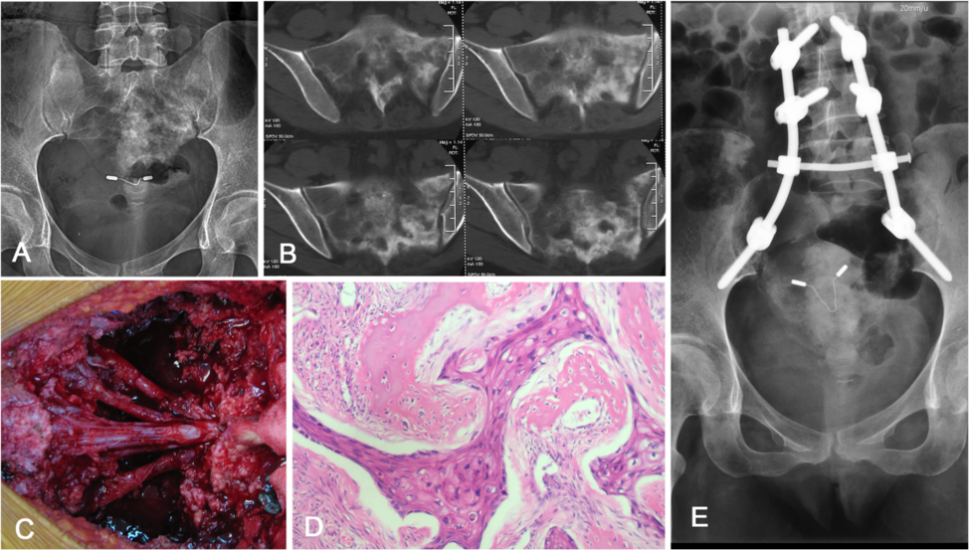
**The patient was diagnosed for cervical cancer (Number 14).** X-ray **(A)** and computed tomography (CT) **(B)** showed a large sclerotic lesion involving the sacrum. Intraoperative picture **(C)** demonstrated sacral nerve roots preserved after resection of metastatic lesion. Pathological examination revealed squamous cell carcinoma **(D)** (hematoxylin and eosin (H&E) stain, ×100). Postoperative x-ray **(E)** showed screw-rod system reconstruction.

Indications for orthopedic surgery [15] included intractable pain in ten patients (56%), impending or pathological fracture in five (28%) patients, and spinal cord compression in three patients (16%). Wide resection consisting of *en bloc* removal of the bone lesion with an envelope of normal tissue and reconstruction was done in five patients. Intra-lesional curettage followed by internal fixation was performed in 13 patients (Figure 1). Palliative decompression for spinal cord compression was performed in four patients. All the patients received bisphosphonate treatment monthly after surgery.

The primary functional outcome was defined as improvement in the specific pain and performance status for which the surgical intervention was performed. Patients were evaluated preoperatively and every three months after surgery. A visual analog pain scale (VAS) was used to evaluate the degree of pain both before and after surgery. An Eastern Cooperative Oncology Group (ECOG) performance status was evaluated both pre- and post-operatively. This measured functional performance on a scale from 0 to 4, where 0 represents normal activity and 4 signifies complete bed rest. In order to minimize the impact of cancer recurrence on the primary outcome of pain improvement and performance status, we focused the analysis on immediate pre-operative assessment and that occurring in the initial 3 months after surgical intervention.

Statistical analysis was performed using Statistical Package for the Social Science (SPSS) software version 20.0 (SPSS Inc, USA). A comparison of parametric outcome data was performed with a paired t test. A Kaplan-Meier survival method was used to estimate patient survival.

## Results

**Figure 2:**
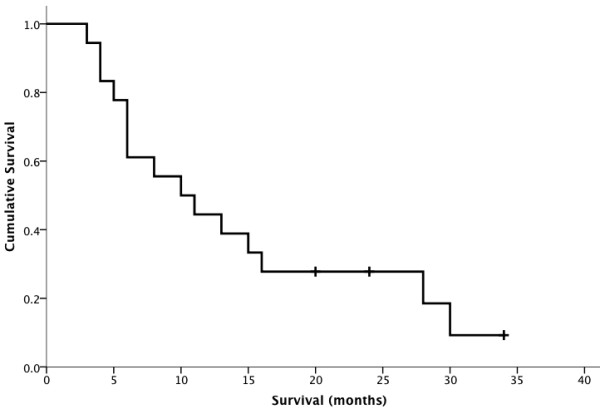
**The Kaplan-Meier survival curve showed patients’ survival after bone metastases from gynecological malignancies.** The median survival time from diagnosis of bone metastasis was 10.0 months (95% confidence interval (CI), 3.8 to 16.2 months).

At completion of data collection, 15 patients were dead of primary disease, and 2 patients were alive without evidence of disease recurrence. One patient (patient 16) developed pulmonary metastasis 19 months after femur surgery and is alive with disease. New bone lesions occurred in two patients (patient 5 and 8) during the follow-up and no simultaneous surgical intervention was performed. The survival curve for the 18 patients is shown in Figure 2. The median survival time following diagnosis of bone metastasis was 10.0 months (95% CI, 3.8 go 16.2 months). The estimated 1-year survival was 44.4%. The eight patients with endometrial carcinoma had a median survival of 10.0 months (95% CI 0 to 22.5 months), patients with cervical cancer 6.0 months (95% CI 4.9 to 7.1 months), and ovarian carcinoma 8.0 months (95% CI 4.8 to 11.2 months).

The mean VAS pain scale score was 5.8 (SD 1.5) preoperatively compared with 2.1 (SD 1.9) 3 months after surgery. There was a significant improvement in pain 3 months after surgical intervention (*P* <0.001). Pain relief was observed in 17 patients (94.4%). The mean pre- and post-operative ECOG performance status grades for all 18 patients were 3.1 (SD 0.9) and 2.3 (SD 1.1), respectively. Post-operatively, five patients (27.8%) performed well (ECOG performance status of 1 or 0) compared with only one (5.6%) pre-operatively. The paired samples t-test showed a statistically significant improvement in ECOG performance status (*P* = 0.02). Improvement in performance status was seen in 12 of the 18 patients (66.7%). The performance of two patients remained unchanged and four (22.2%) deteriorated.

There were a total of 6 complications among the 18 patients, for an overall complication rate of 33.3%. Two lesions (patient 6 and 12) recurred following curettage, neither of these patients received additional radiotherapy. Two patients developed an infection or wound dehiscence; one required debridement. A cerebrospinal fluid leak was diagnosed in patient 8, and was managed conservatively. Femoral vein thrombosis occurred in one patient.

## Discussion

Recurrent gynecologic cancers are not confined to the abdominal peritoneal cavity. These diseases exhibit a significant potential for distant metastases, even at early stages, as evidenced by the identification of bone metastases in cervical cancer patients with suspected clinical stage 2B disease. In a study of hematogenous metastases in patients with FIGO Stage 1 or 2 endometrial carcinoma, 134 metastatic sites were identified in a total of 110 patients, and bone involvement was observed in 23 patients [16].

Chang *et al*. [17] described a case of FIGO stage IA epithelial ovarian cancer with bone as the first site of recurrent disease. The treatment of patients with bone metastases should be tailored to the individual patient and based on factors such as age, performance status, presence of simultaneous metastasis, and site of bone affected. The primary goal of surgical intervention is to eliminate or alleviate pain and preserve quality of life, with a possible prolongation of life [18]. There are few previous reports detailing both quality of life parameters and functional outcomes following surgical treatment for recurrent gynecological malignancies presenting as bone metastasis.

Two mechanisms for osseous metastasis in gynecologic cancer have been described [19]. Direct invasion into bone from loco-regional or distant soft tissue tumor masses is one mechanism, and hematogenous spread through the systemic circulation or the Batson venous plexus is another. In the current series, 66.7% of metastatic lesions were in the pelvic girdle or spine. The spread to lower limb bones is thought to occur primarily via venous retrograde flow of tumor emboli [20,21].

In a study of 377 patients, 25% of patients with loco-regionally restricted gynecologic cancer were found to have disseminated tumor cells in bone marrow at diagnosis. Loizzi *et al*. [22] reviewed 21 endometrial cancer patients with bone metastasis from 20 published reports. They found the median interval between the detection of endometrial cancer and bone metastasis to be 21 months, similar to the mean interval of 21.8 months identified in the current study. An analysis on bone metastasis in cervical cancer patients over a 10-year period showed the median duration from diagnosis of cervical cancer to bone metastasis was 16 months and the median survival was 7 months following intervention for this subgroup of patients [6]. In a retrospective review of 103 ovarian cancer cases, four patients had bone involvement with a median survival of 7.5 months [23]. These survival results mirror our experience, with median survival of 10 months for endometrial cancer, 8 months for ovarian cancer, and 6 months for cervical cancer.

Estimating survival in patients with bone metastases is imperative in surgical treatment planning [8,24,25]. However, there are challenges inherent to predicting survival [10]. Three-month and 12-month time points were considered to be useful discriminators for surgical decision-making. It is well accepted that survival less than 3 months is a relative contraindication to surgical intervention, with specific exceptions such as acute spinal cord compression. Shorter life expectancies (3 to 12 months) are thought to warrant less invasive stabilization procedures, while longer life expectancies (>12 months) warrant more durable reconstruction, which is usually associated with operative morbidity and a longer period of convalescence [26]. Recently, uncemented fixation or tantalum trabecular metal implants have been used to achieve more durable reconstructions in metastatic conditions [27].

The ECOG performance status was found to be a significant prognostic factor in prior studies investigating management of metastatic bone disease. The simplicity of use and reproducibility make it a valuable index for prognostication [10,28]. A statistical study based on Bayesian methods identified ECOG performance status and presence of visceral metastases as the most important in developing surgeon’s estimate of survival [26]. The prognosis of bone metastases from breast cancer or prostate cancer is measurable in years. In contrast, the median survival time form the diagnosis of advanced lung cancer is typically measured in months [29,30]. Due to the rarity of bone metastasis from gynecologic malignancies, the prognosis of these patients after surgical treatment has not been well established.

The oncological objective of excising metastatic tumor of bone is the achievement of local tumor control. However, skeletal-related events involving pathological fractures, spinal cord compression and a need for surgery and/or radiotherapy may potentially have detrimental effects on survival and quality of life. In this study, surgical intervention for the treatment of bone metastases resulted in a significant improvement in pain as well as ECOG performance status. When a cure is not achievable, palliation of symptoms and improvements in quality of life should be a priority.

**Figure 3:**
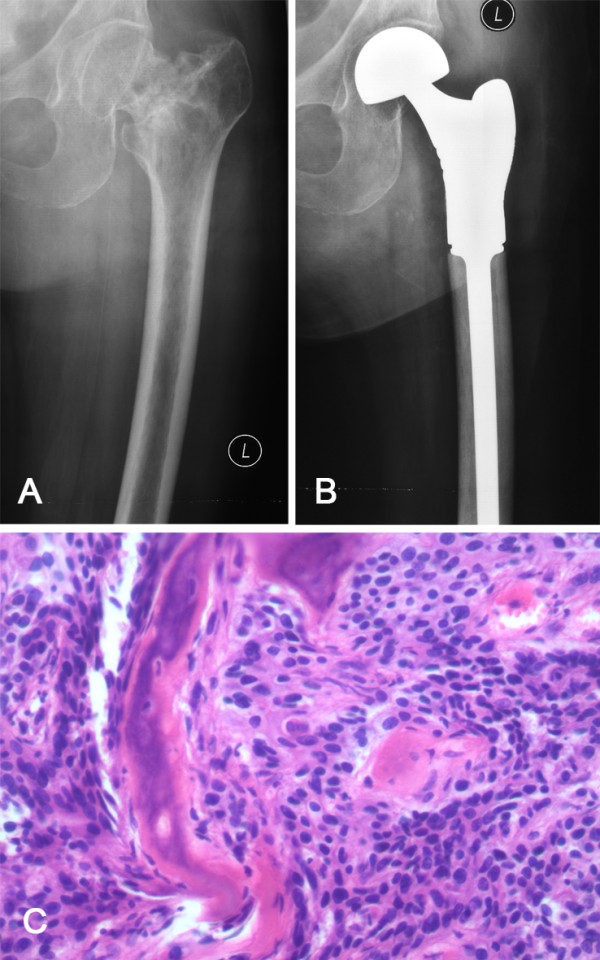
**Representative case showed pathological fracture in femoral neck of patient number 17.** The preoperative X ray showed the pathological fracture **(A)**.A proximal femoral endoprosthesis was used to reconstruct the bone defect **(B)**. Histological appearance **(C)** of the lesion featuring infiltration by poorly differentiated squamous carcinoma cells (hematoxylin and eosin (H&E) stain, ×200). Immunohistochemically, the tumor was positive for 34βE12 +, CK5/6 + and p63 +.

Intralesional curettage can decrease rates of local recurrence, avoiding the need for extensive resection and reconstruction. More aggressive local and systemic treatment is advocated for improved local control [28]. Additionally, current systemic treatments and improvements in supportive care have translated into prolonged survival following management of bone metastases. These factors have compelled more aggressive operative procedures, although indications remain controversial. *En bloc* resection may be more appropriate when advanced disease precludes internal fixation, or when the metastatic disease is limited to a solitary bony deposit. Five patients received *en bloc* resection in the current study, three of which were for proximal femoral lesions (Figure 3). Outcomes of internal fixation for this site are often unfavorable, with high nonunion rate (65%), high local recurrence rate (48%), and high implant failure rate (23%) [31-33]. Use of endoprostheses demonstrates a lower mechanical failure rate and a higher rate of implant survivorship [31].

It is acknowledged that there are limitations to this study. The present series is a retrospective review and therefore susceptible to the same limitations and biases inherent to all retrospective studies. There is no control group and it is unlikely that ethical approval would be given for a no-treatment or no-surgical intervention group. Nonetheless, this paper is among the largest series published to date on surgical treatment for bone metastases in gynecological tumors and is the first to document quality of life and functional parameters after treatment.

## Conclusions

In conclusion, the management and counseling of patients with gynecologic cancers metastatic to bone is difficult. Specifically, prognosis of gynecological cancer patients with bone metastasis is poor. Nonetheless, following surgical intervention, improvements in quality of life have been described. Given the small number of patients and heterogeneity of our study population, no definite conclusions can be drawn. A prospective, multi-institutional study may allow for collection of more comprehensive data, helping guide gynecologic oncologists in the counseling of such patients.

## Abbreviations

AWD: alive with disease; CC: cervical cancer; DOD: died of disease; EC: endometrial carcinoma; EC^❖^: uterine leiomyosarcoma; ECOG: Eastern Cooperative Oncology Group; IP: intractable pain; NED: no evidence of disease; OC: ovarian carcinoma; PF: pathological fractures; Chemo: Chemotherapy; RT: radiotherapy; SCC: spinal cord compression; Surg: surgery; VAS: visual analog scale.

## Competing interests

The authors declare that they have no competing interests.

## Authors’ contributions

TJ, BH and WG conceived and designed the study, analyzed and interpreted data, and drafted and revised the manuscript. YW collected the follow-up results and medical records. KS reviewed the pathology slides. RE reviewed the gynecological treatment document and revised the manuscript. All authors read and approved the manuscript.
